# Construction of Inverse–Opal ZnIn_2_S_4_ with Well–Defined 3D Porous Structure for Enhancing Photocatalytic H_2_ Production

**DOI:** 10.3390/nano14100843

**Published:** 2024-05-11

**Authors:** Yiyi Xie, Zhaohui Wu, Sifan Qi, Jiajun Luo, Shuang Pi, Huanghua Xu, Shumin Zhang, Difa Xu, Shiying Zhang, Xianfeng Yang

**Affiliations:** 1 College of Materials Science and Engineering, Changsha University of Science & Technology, Changsha 410114, China; 2 Hunan Key Laboratory of Applied Environmental Photocatalysis, Changsha University, Changsha 410005, Chinazsmstudy@foxmail.com (S.Z.);

**Keywords:** ZnIn_2_S_4_, inverse opal, slow photon effects, hydrogen evolution

## Abstract

The conversion of solar energy into hydrogen using photocatalysts is a pivotal solution to the ongoing energy and environmental challenges. In this study, inverse opal (IO) ZnIn_2_S_4_ (ZIS) with varying pore sizes is synthesized for the first time via a template method. The experimental results indicate that the constructed inverse opal ZnIn_2_S_4_ has a unique photonic bandgap, and its slow photon effect can enhance the interaction between light and matter, thereby improving the efficiency of light utilization. ZnIn_2_S_4_ with voids of 200 nm (ZIS–200) achieved the highest hydrogen production rate of 14.32 μ mol h^−1^. The normalized rate with a specific surface area is five times higher than that of the broken structures (B–ZIS), as the red edge of ZIS–200 is coupled with the intrinsic absorption edge of the ZIS. This study not only developed an approach for constructing inverse opal multi–metallic sulfides, but also provides a new strategy for enriching efficient ZnIn_2_S_4_–based photocatalysts for hydrogen evolution from water.

## 1. Introduction

The growing global concern regarding the energy crisis and environmental pollution has sparked significant interest within the scientific and industrial communities in developing clean and sustainable energy alternatives to traditional fossil fuels [1,2]. Solar energy is an inexhaustible, clean and renewable natural resource, and hydrogen can serve as an energy carrier for solar energy with a high energy conversion efficiency and zero carbon, and the only byproduct is water. As is well known, photocatalysis is a potential economical, environmentally friendly, simple and efficient method that utilizes solar energy upon catalysts to promote the decomposition of water for generating green hydrogen [3,4,5,6,7,8]. However, some factors lead to low water splitting efficiency in the photocatalytic process, such as limited light absorption, excessive charge carriers’ recombination and interface reaction. Among them, the utilization of light over the photocatalyst directly determines the upper limit of the process, which can be significantly enhanced by different methods, such as structural control [9], element doping [10], defect engineering [11] and heterojunctions [12]. Specifically, the construction of inverse opal photocatalysts with three–dimensional ordered macropores (3DOM), as an efficient strategy for enhancing light absorption, has garnered substantial interest from researchers.

As known, 3DOM possesses unique structural and optical properties, including a well–organized and uniform macroporous framework, a significant specific surface area, and a slow photon effect, contributing to enhancing the light absorption efficiency. For example, a hierarchical microporous structure with an open interconnection greatly improves the transport and diffusion efficiency of reactive species [13,14]. Then, a periodic ordered structure makes it produce a photonic bandgap and can slow the photon propagation in photonic crystals (i.e., a slow light effect), which can be absorbed effectively by photonic crystals, thus producing more photo–induced carriers [15]. Therefore, the 3DOM photocatalyst also exhibits a superior carriers’ separation and mass transfer, except for a unique light absorption capacity [16,17,18,19,20,21,22,23,24]. It is foreseeable that the 3DOM semiconductor is a promising kind of photocatalyst. Regretfully, the reported IO photocatalysts are predominantly metal oxides, such as titanium oxide [25], tungsten oxide [18] and indium oxide [26], which usually have a wide bandgap and respond to UV light. Notably, due to the fact that the light utilization of the IO structure is mainly based on specific light with a slow effect in the red edge of the photonic crystal, it exhibits the best light absorption when this partial slow light highly overlaps with the band–edge region of the semiconductor. Thus, the IO structure cannot expand the range of the light absorption, but it enhances specific light utilization in the light response range of the semiconductor [27,28,29]. As a result, the total number of the absorbed photons would be limited by the narrow light response in the above–mentioned semiconductors. Hence, developing an IO structure of semiconductor with a narrow bandgap would be an effective strategy for further improving light utilization, but this aspect’s study is scarce.

As a narrow bandgap semiconductor, ZnIn_2_S_4_ has tunable bandgap ranges from 2.06 to 2.85 eV, giving a strong response in the visible light region [30,31,32]. Meanwhile, ZnIn_2_S_4_ has been proven as a promising photocatalyst for H_2_ production, carbon dioxide fixation/conversion and degradation of pollution [33,34,35]. However, ZIS photocatalysts’ development is also limited by the low utilization rate of the responding light and high recombination rate of photogenerated carriers [36,37]. Coincidentally, the unique merits of the IO structure could make up for the above shortcomings. On the one hand, the slow photon effect can enhance the interaction between light and matter to improve the light absorption efficiency. On the other hand, the uniform macroporous framework can shorten the migration distance of carriers to reduce carriers’ recombination [38,39]. It is not hard to foresee that IO ZIS would be an ideal candidate photocatalyst.

Herein, IO ZIS with different pore sizes was successfully constructed using the soft template method. Among the inverse opals with varying voids, the ZIS with an inverse opal structure exhibits superior hydrogen production performance compared to the ZIS with a disrupted inverse opal structure, after normalizing the specific surface area. Among the as–prepared samples, ZIS–200 exhibited a better hydrogen production rate of 1431.84 μmol g^−1^ h^−1^, mainly due to the overlap between the red edge of the bandgap of ZIS–200 and the electronic bandgap edge of the ZIS. Therefore, the IO structure of the ZIS photocatalyst provides new insights for designing and fabricating highly efficient ZIS–based photocatalysis, as well as expanding the range of narrow–bandgap photonic crystal catalysts.

## 2. Materials and Methods

### 2.1. Materials

Polyvinyl pyrrolidone (PVP K30, (C_6_H_9_NO)_n_) was purchased from Tianjin Chemical Reagent Co., Ltd. (Tianjin, China). Indium chloride tetrahydrate (InCl_3_·4H_2_O), H_2_O_2_ (98%), sulfuric acid (98%), H_2_O_2_ (98%), ethanol (99.5%), styrene (St, 99%), N–N–Dimethylformamide and chloroplatinic acid hexahydrate (H_2_PtCl_6_·6H_2_O, 98.5%) were purchased from Sigma-Aldrich (Shanghai, China), Granular sodium hydroxide (NaOH), zinc chloride anhydrous (ZnCl_2_), potassium persulfate (KPS, 95%) and thioacetamide (C_2_H_5_NS, TTA) were purchased from Sinopharm Chemical Reagent Co. (Shanghai, China) and used without further purification.

### 2.2. Synthesis of Polystyrene (PS) Spheres

Monodisperse PS colloids with different diameters (d = 120, 180, 200 and 220 nm) were synthesized using the soap–free emulsion polymerization method as described in the literature with minor changes [28]. First, the styrene monomer is washed with NaOH solution (10%) and deionized water to remove the inhibitors 3 times. Briefly, some St and 0.4 g of PVP were added at room temperature to 50 mL of deionized water in a 250 mL three–neck round–bottom flask under nitrogen protection. The mixture solution was then stirred at about 300 rpm for 30 min, gradually increasing the mixing temperature to 70 °C until the temperature remained unchanged. Then, 0.1 g of KPS dissolved in 10 mL of deionized water was added to polymerize the reaction, and kept at this temperature (70 ± 3 °C) for 24 h. The above–mentioned product was obtained to remove residual styrene and PVP by centrifugal washing with deionized water and alcohol three times.

### 2.3. Synthesis of 3DOM ZnIn_2_S_4_

**Scheme 1:**
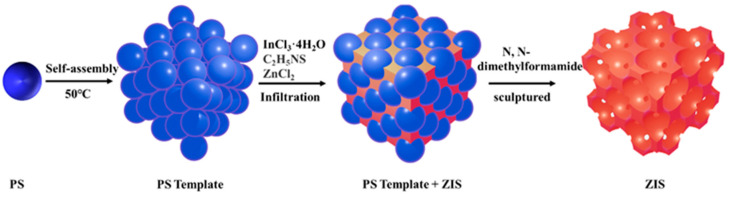
Schematic diagram of IO–ZIS preparation.

The glass slides, which were pretreated to make their surface hydrophilic by immersing them in piranha solution (H_2_O_2_ and H_2_SO_4_ at the ratio of 3:7) for 12 h, were self–assembled by adding monodisperse polystyrene spheres that were diluted with deionized water to a concentration of 5 vol % and ultrasonic treatment form PS colloidal crystal templates with face–centered cubic (FCC) arrays. Then, 0.293 g of InCl_3_·4H_2_O, 0.225 g of C_2_H_5_NS and 0.068 g of ZnCl_2_ were dissolved in 10 mL of deionized water and stirred rapidly with a magnetic beater to obtain a homogeneous solution. Subsequently, the PS templates were dipped into the aforementioned mixed solution at room temperature for 20 min, and then the glasses were taken from beakers, which were repeatedly filled five times with a vacuum drying oven to ensure that the precursor solution was completely filled in the holes, and then the obtained samples were heated in a vacuum drying oven at 80 °C for 2 h. Finally, the PS spheres were removed by immersing in N,N-dimethylformamide for 12 h, and the inverse opal structure of the ZIS was obtained. The samples were named according to the void diameter, e.g., ZnIn_2_S_4_ with voids of 120 nm was denoted as ZIS–120. In addition, the IO–ZIS on the glass substrate was ground to break the inverse opal structure as a reference sample (labeled as B–ZIS). The preparation procedure is illustrated in Scheme 1.

### 2.4. Characterization

The morphology and structure of the 3DOM ZIS were observed by field–emission scanning electron microscopy (SEM, JEOL, JSM–IT800, Tokyo, Japan) and transmission electron microscopy (TEM, JEOL, JEM–F200, Tokyo, Japan). The crystal structure of the samples was examined by powder X-ray diffraction (XRD, SmartLab9kw, Karlsruhe, Germany) with a Cu anode X-ray tube (λ = 1.54056 Å), the scanning range of 2θ was from 10° to 80° and the scanning rate was 10° min^−1^. The UV–Vis absorption spectra were collected by a SHIMADZU UV–Vis (UV–2600, Shanghai, China) spectrophotometer in the range of 200–800 nm with BaSO_4_ as the reference. The X-ray photoelectron spectroscopies (XPS) of the photocatalyst were collected in an instrument (Thermo ESCALAB 250XI, Waltham, MA, USA) assisted with a Mg–Kα source. The Brunauer–Emmett–Teller (BET) surface areas of the as–prepared catalyst were obtained through the Quantachrome Autosorb–iQ (Boynton Beach, FL, USA).

All PEC measurements were performed in a standard quartz three–electrode battery with a Pt foil as the counter electrode, Ag/AgCl as the reference electrode and a photoanode prepared using IO ZIS and B–ZIS nanoparticles on the FTO substrate as the working electrode in 0.5 M Na_2_SO_4_. The Mott–Schottky plots were measured with a bias potential ranging from −0.7 V to 0.7 V (vs. Ag/AgCl). The electrochemical impedance spectroscopy (EIS) was performed on an electrochemical workstation (CHI660E, Shanghai, China) in the frequency range from 100 kHz to 10 mHz in a standard three–electrode system using the as–fabricated photocatalyst films as the working electrodes, a Pt plate electrode as the counter electrode and Ag/AgCl (saturated in KCl) as the reference electrode. The transient photocurrent response vs. time was measured in 0.5 mol/L Na_2_SO_4_ electrolyte with light on/off cycles for different ternary samples under visible light irradiation at a bias of 0 V vs. Ag/AgCl.

### 2.5. Photocatalytic H_2_ Production Tests

Typically, 0.01 g of the sample is immersed in an aqueous solution containing 0.2 M lactic acid as the sacrificial agents. The mixture is sealed in a quartz vessel and stirred during photoreaction. Pt nanoparticles were photo–deposited on the surface of the ZnIn_2_S_4_ photocatalysts, thereby creating cathodic sites for photocatalytic hydrogen evolution. After degassing, using a 300 W Xenon lamp with a cutoff filter (λ ≥ 400 nm) as a visible light source, the vessel was under irradiation of visible light. The gas products were analyzed periodically by an Agilent 7900 A gas chromatograph (GC) with a thermal conductivity detector (TCD).

## 3. Results and Discussion

### 3.1. Morphology and Crystal Structure

**Figure 1:**
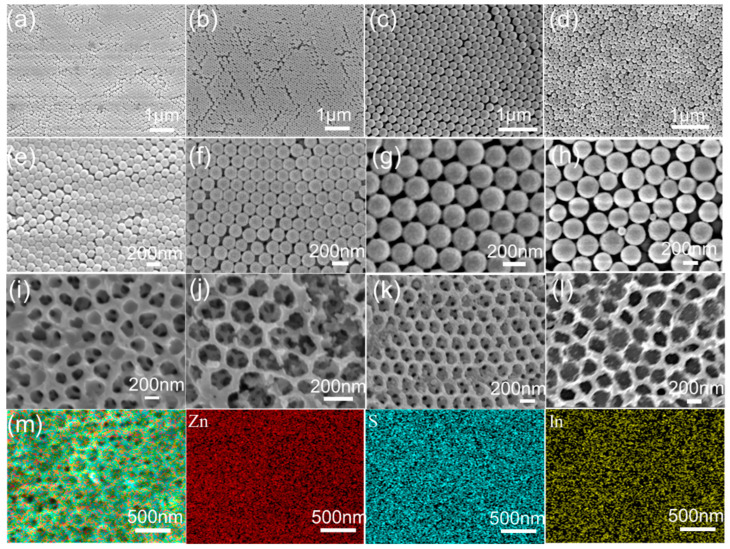
SEM images of opals with 120 (**a**,**e**), 160 (**b**,**f**), 200 (**c**,**g**) and 220 (**d**,**h**); SEM images of inverse opals with 120 (**i**), 160 (**j**), 200 (**k**) and 220 (**l**); and EDX mapping images of ZIS–200 (**m**).

SEM images can be employed to characterize the morphologies of as–prepared samples, so the morphology of the initial PS sphere arrays, and final pore structure of the inverse opal ZIS are shown in Figure 1. Of course, microscopic observation confirmed the formation of an ordered and uniform structure of opal and inverse–opal materials; Figure 1a,e, Figure 1b,f, Figure 1c,g and Figure 1d,h correspond to PS microspheres with sizes of approximately 120, 160, 200 and 220 nm, respectively, while the high–ordered and uniform pores shown in Figure 1i, Figure 1j, Figure 1k and Figure 1l correspond to ZIS–120, ZIS–160, ZIS–200 and ZIS–220, respectively. Therefore, it has been shown that in the ordered structure of the opal and IO–ZIS, the smoothest surface and least cracks were observed on the IO–ZIS sample. Finally, a hierarchical, dense and continuous IO–ZIS structure was obtained. As a result, it can be observed that IO–ZIS exhibits four types of pores with average sizes of 120, 160, 200 and 220 nm, which interconnect to form the intended inverse opal structure. However, many cracks were observed in IO–ZIS samples at lower magnification, which may be due to the condensation and decomposition of the precursor, as well as the interaction between the precursor and the PS sphere during the infiltration and calcination process, which may lead to the collapse of the template [40]. Its EDX energy spectrum is depicted in Figure 1m, where it can be observed that Zn, In and S are uniformly distributed in the structure. As shown in Figure S1, it can be seen from the EDX analysis that the Zn, In and S are mainly from samples, and their atomic ratio is close to 1:2:4. The structure of synthetic inverse opal will not affect the element distribution of the ZIS. Therefore, the EDX element analysis of the B–ZIS is shown in Figure S2. It can be seen that the element Zn In and S is evenly distributed in the sample, and the atomic ratio is basically close to that of the IO–ZIS. Additionally, it can be seen that the Zn atomic ratio of the IO–ZIS and B–ZIS is low, which is mainly due to certain defects in the ZnIn_2_S_4_ during the synthesis process, resulting in the low content of Zn.

**Figure 2:**
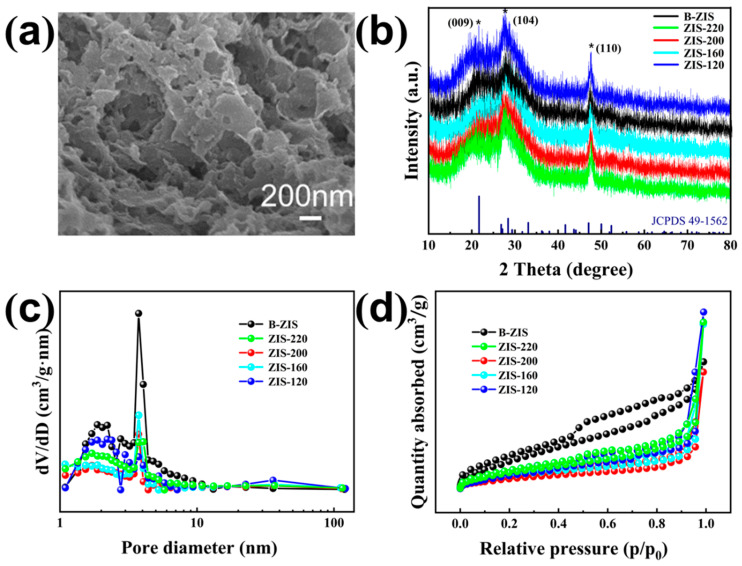
SEM images of B–ZIS (**a**); XRD patterns of ZIS–120, ZIS–160, ZIS–200, ZIS–220 and B–ZIS samples (**b**); pore–size distribution curves (**c**) and N_2_ adsorption–desorption isotherms of B–ZIS, ZIS–220, ZIS–200, ZIS–160 and ZIS–120 composites (**d**).

To provide a better contrast with the inverse opal–structured ZIS, the structure of ZIS–200 was disrupted to eliminate the inverse opal characteristics, as demonstrated in Figure 2a, which obviously makes more surfaces exposed, whether the optical characteristics and electrochemical properties were affected or not. Further characterizations of the prepared samples were conducted using X-ray diffraction (XRD), pore–size distribution curve isotherms and N_2_ adsorption–desorption to determine the crystal phase structure, specific surface area, and pore–size distribution, providing a comprehensive description of the materials. The crystal structure of the as–prepared photocatalyst can be recorded in XRD patterns, as shown in Figure 2b, and all the characteristic peaks of these samples in the XRD patterns are indexed to hexagonal ZnIn_2_S_4_. However, the lower intensity of all the peaks suggests a lower crystallinity due to a low temperature. The diffraction peaks of the 2θ at 21.6°, 28.3° and 47.9° correspond to the (009), (104) and (110) planes of the hexagonal ZnIn_2_S_4_ (JCPDS No. 49–1562), respectively, indicating that the IO structure would not alter the basic crystal structure of the as–prepared samples. To verify the pore–size distribution of the material, as depicted in Figure 2c, the pore–size distribution of the five samples was ranged from 2 nm to 50 nm, and N_2_ adsorption–desorption was used to study the b–ZIS, ZIS–120, ZIS–160, ZIS–200 and ZIS–220 samples in Figure 2b. The adsorption–desorption isotherms of the materials all exhibited the IV–type isotherm model. At higher relative pressures, a clear hysteresis loop was observed, indicating the presence of a mesoporous structure in the materials [41]. From Table 1, it is evident that the specific surface area of B–ZIS (105.21$\;m^{2}\cdot g^{-1}$) has significantly increased in comparison to ZIS–120 (51.79 $m^{2}\cdot g^{-1}$), ZIS–160 (46.98 $m^{2}\cdot g^{-1}$), ZIS–200 (36.59 $m^{2}\cdot g^{-1}$) and ZIS–220 (57.56 $m^{2}\cdot g^{-1}$), which can be attributed to the disruption of the inverse opal structure, leading to the loss of the pore structure and exposing a greater specific surface area. Combined with the above–mentioned SEM analysis of ZIS–220, the uniformity is insufficient when the particle size is large. The non-uniform PS spheres have a greater compactness, and the specific surface area exposure is greater after the template is removed, thus increasing the specific surface product brought by the small spheres, which makes the specific surface of ZIS–220 larger.

**Figure 3:**
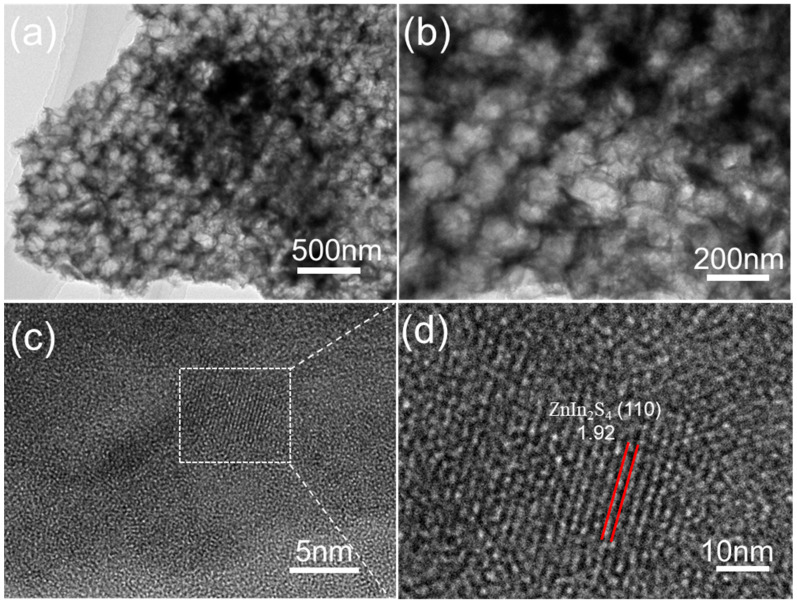
TEM images of ZIS–200 (**a**,**b**); HRTEM images (**c**,**d**).

**Figure 4:**
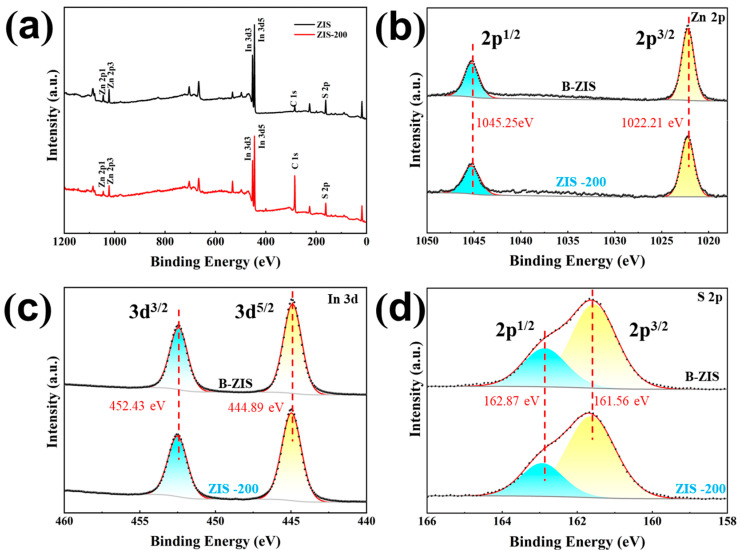
XPS spectra of B–ZIS and ZIS–200: (**a**) survey, (**b**) Zn 2p, (**c**) In3d 2p and (**d**) S 2p.

The successful construction of the three–dimensional macroporous ZIS–200 of the sample was confirmed by the TEM images in Figure 3a,b, which is consistent with that depicted in the SEM images. Additionally, the high–resolution TEM (HRTEM) image in Figure 3c,d presents lattice stripes with a plane spacing of about 1.92 nm, which are attributed to the (110) crystal planes of ZIS, further confirming the maintained 3D ZIS structure. In addition, X-ray photoelectron spectroscopy (XPS) spectra were measured to investigate the surface chemical state and chemical composition of B–ZIS and ZIS–200. As shown in Figure 4a, the XPS spectrum of B–ZIS and ZIS–200 shows the peaks of only the Zn, In and S elements in the sample, which is also consistent with the elemental mapping. Figure 4b demonstrates the Zn 2p high–resolution XPS spectra, and two characteristic peaks arising from Zn^2+^ 2p^3/2^ and Zn^2+^ 2p^1/2^ are located at about 1022.21 eV and 1045.25 eV, respectively. Figure 4c shows two characteristic peaks located at 444.89 eV and 452.43 eV corresponding to In^3+^ 3d^5/2^ and In^3+^ 3d^3/2^, respectively. Figure 4d shows two characteristic peaks at 161.56 eV and 162.87 eV that have arisen from S 2p^3/2^ and S 2p^1/2^, respectively, corresponding to S^2−^ ions [37]. No significant shift of these characteristic peaks was observed in b–ZIS and ZIS–200, indicating that the introduction of the inverse opal structure did not alter the chemical state of the ZIS.

### 3.2. Optical Properties

**Figure 5:**
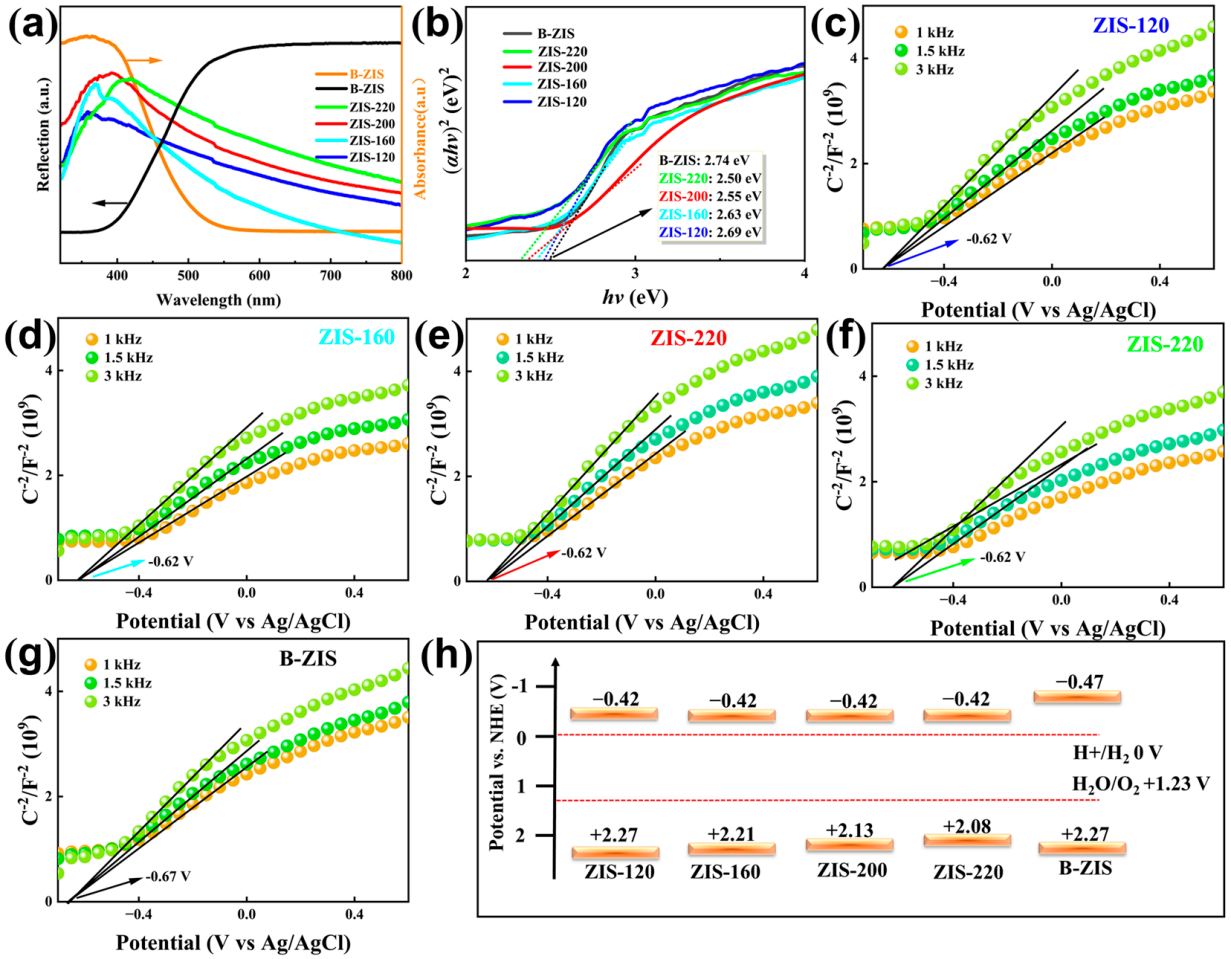
UV–vis DRS spectra of B–ZIS, ZIS–120, ZIS–160, ZIS–200 and ZIS–220 samples (**a**); plots (*αhν*)^2^ (eV)^2^ for the bandgap energies of the photocatalysts (**b**); M–S curves of as–prepared photocatalysts with 1, 1.5 and 3 kHz (**c**–**g**), respectively; schematic diagram of energy band (**h**).

To detect the slow photon effect and light absorption capacity of as–fabricated samples, a UV–vis diffuse reflectance spectroscopy (DRS) measurement was performed, as shown in Figure 5a. According to different sizes of the ZIS inverse opal, it can be observed that compared with ZIS, the optical absorption edge shifted to a higher energy with the increase of the void size of the inverse opal, which corresponds to the Bragg diffraction equation. Furthermore, ZIS–200 exhibited an apparently increased light response range relative to other samples, which could be attributed to the slow photon effect caused by the unique IO structure. The inverse opal structure has obvious reflection peaks, which are photon bandgaps, but the reflection peaks disappear when the inverse opal structure of the ZIS is destroyed. Additionally, the photonic bandgaps of ZIS photonic crystals of different sizes are approximately 355, 378, 415 and 436 nm. The bandgap energy (E_g_) of the aforementioned samples was determined from Figure 5b, and the calculated bandgap values for ZIS–120, ZIS–160, ZIS–200, ZIS–220 and B–ZIS are 2.69, 2.63, 2.55 2.50 and 2.74 eV, respectively, using the formula $(\alpha h\nu {)}^{1/n}=A\left(h\nu -E_{g}\right)$. Among the above photocatalysts, with the increase of pore size, ZIS–120 shows a higher bandgap value due to the small–size effect, while B–ZIS shows a higher bandgap value from the new surface exposed, resulting in defects that affect the band position of the ZIS. As shown in Figure 5c–g, Mott–Schottky plots were used to measure the flat–band potentials of the as–prepared photocatalysts, which was analyzed by using the Mott–Schottky (M–S) measurement at the frequencies of 1, 1.5 and 3 kHz. As can be seen from Figure 5c–g, all of the E_fb_ of IO–ZIS are −0.62 V vs. Ag/AgCl, but B–ZIS showed the highest flat–band position of 0.67 vs. Ag/AgCl, mainly due to a large number of defective surfaces, which may be caused by removing the template in the same way as IO–ZIS or during grinding. Additionally, it was particularly noteworthy to recognize that n–type semiconductors typically exhibit a conduction band potential (E_CB_) approximately equal to E_fb_. Therefore, the conduction potentials (CB) of IO–ZIS transformed into the normal hydrogen electrode (NHE) were −0.42 V vs. NHE, and the corresponding valence band (VB) potentials were 2.27, 2.21, 2.13, 2.08 and 2.27 V vs. NHE, respectively, which were nearly identical to the results calculated by using the equation of E_VB_ = E_CB_ + E_g_. Obviously, the energy band diagram of the as–prepared samples is shown in Figure 5h, and the conduction potential of the as–prepared photocatalyst is higher than the potential of H+/H_2_ (0 V), providing a stronger reduction ability for H_2_ production. Among the IO–ZIS photocatalysts, the flat–band potential of the IO–ZIS did not change due to the same material. The light absorption of the photocatalysts is different according to the size of the gap. ZIS–220 has a larger gap, and the size of the ZIS between the gaps becomes smaller. Therefore, compared with other IO–ZISs, ZIS–220 has a smaller bandgap value. However, B–ZIS is ground by ZIS–200, which will expose more surfaces and defects, thus changing its band position, with more a negative conduction band and a corrected valence band position. Hence, the as–prepared samples demonstrate an enhanced visible light absorption efficiency, showing promising potential for photocatalytic hydrogen production.

### 3.3. Photocatalytic Property for H_2_ Production

**Figure 6:**
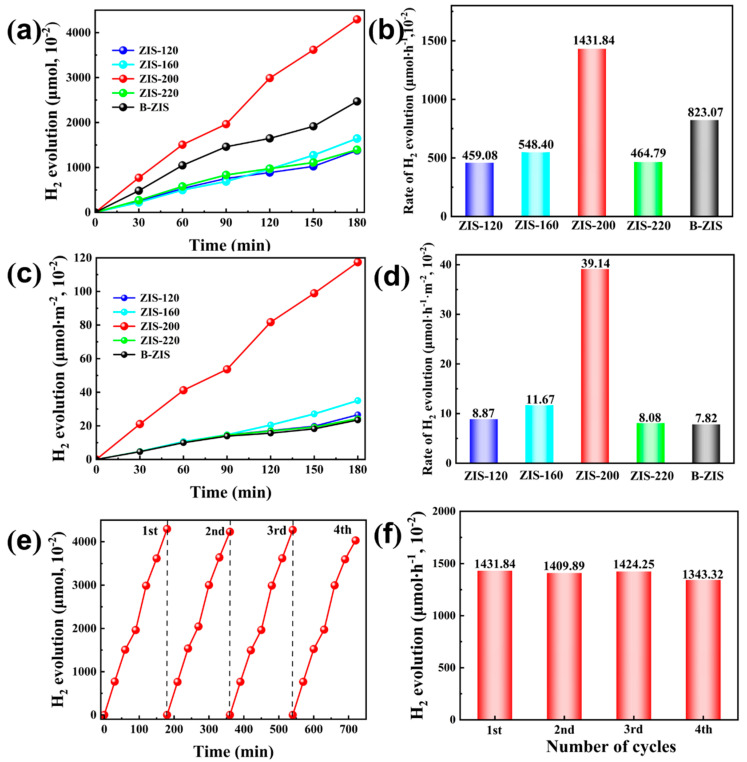
Photocatalytic H_2_ production time–yield plots of as–prepared photocatalysts under light irradiation (**a**); photocatalytic H_2_–generation ability of as–prepared photocatalysts under light irradiation (**b**); H_2_ production time–yield plots of as–prepared photocatalysts under light irradiation by the normalized rate with specific surface area (**c**); H_2_–generation ability of as–prepared photocatalysts by the normalized rate with specific surface area (**d**); cycling experiments for ZIS–200 (**e**,**f**).

The photocatalytic efficiencies of the different photocatalysts were evaluated in the presence of sacrificial reagent lactic acid through hydrogen production under visible irradiation. From Figure 6a,b, all the samples exhibited gradually increasing hydrogen production performance when prolonging the irradiation time. It is also worth noting that ZIS–200 performed superior hydrogen evolution (14.32 μmol h^−1^) over other samples, which is almost three times higher than that of ZIS–120. Then, the destruction of the inverse opal structure exposes more surfaces of ZIS, which undergoes a specific surface area normalization treatment for mitigating the impact of the specific surface area on hydrogen production. Particularly, Figure 6c,d presents the hydrogen production data after the specific surface area normalization, with ZIS–200 (0.39 μmol∙m^−2^ h^−1^) continuing to outperform other samples, which is five–fold higher than that of B–ZIS. By comparison, a ZIS hydrogen production test without an IO structure has also been carried out, whose results show that the IO–ZIS photocatalyst has a better H_2_–production capability than the ZIS without an IO structure. This is primarily attributed to the unique periodic dielectric structure of the inverse opal, leading to the formation of a slow light effect, which causes the continuous scattering and reflection of light within the photocatalyst, thereby enhancing the interaction between light and matter. Thus, ZIS–200 is better than the ZIS without an IO structure, as it enhances the carrier migration rate and improves the absorption efficiency of sunlight. It can be seen in Figure 6a that the red–edge slow–photon enhancement for H_2_ generation is much higher than the blue–edge slow photon, and this result seems to be consistent with the traditional concept, that is, the red–edge slow–photon effect has a more practical application in the photocatalytic reaction, because the slow photon directly interacts with the material with the red–edge slow photons concentrated in the low dielectric constant part. As shown in Figure 6e,f, after four cycles, ZIS–200 exhibited no significant decrease in the photocatalytic hydrogen production rate, suggesting that the photocatalytic material possesses a good cycling stability and holds potential for practical applications.

### 3.4. Analysis of Hydrogen Production Mechanism

**Figure 7:**
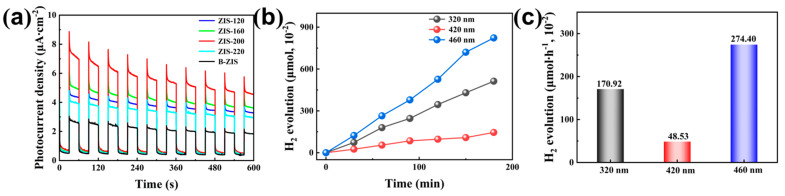
Photocurrent curves of B–ZIS, ZIS–220, ZIS–200, ZIS–160 and ZIS–120 (**a**); hydrogen evolution (**b**) and hydrogen evolution rate (**c**) of photocatalysis under different bandpass wavebands.

In order to examine the photoelectrochemical behavior of all samples subjected to irradiation of visible light, the density of the photocurrent was measured as shown in Figure 7a. During the on/off cycle of irradiation, the photoelectric current of ZIS–200 was notably higher compared to the other samples, indicating its superior capability to generate and transfer photo–induced charge carriers. As the size of the inverse opal structure increases, the photocurrent density of ZIS–200 is the highest, and the photocurrent density of the inverse opal ZIS is related to the size of the opal template, which is higher than that of the B–ZIS. Based on the aforementioned photocurrent, it can be inferred that the ZIS–200 photocatalyst is primarily influenced by the material’s response to light. Thus, it has been confirmed that IO–ZIS plays a pivotal role in the utilization of light in the photocatalytic process. As demonstrated in Figure 7b,c, the study examined the influence of various regions on the hydrogen production performance of ZIS–200, employing bandpass filters to select different bands and verify which side of the photonic bandgap demonstrates superior performance. Hence, Figure 7b shows that the hydrogen production performance of the bandpass in the red–edge region of 460 nm is better than that in the 420 nm photonic bandgap and the 320 nm blue–edge region, and Figure 7c shows that the hydrogen production rates are 1.71, 0.49 and 2.74 μmol h^−1^ for the bandpass wavebands at 320, 420 and 460 nm, respectively. As shown in Figure S3, the B–ZIS wavelength has the best hydrogen–production performance within 320 nm, followed by 420 nm and 460 nm. Because the slow photon effect of this sample disappears, the hydrogen–production performance of B–ZIS is mainly related to the photon energy of the material, which further verifies the utilization of the inverse opal structure for the slow light effect. Therefore, the primary function of the ZIS–200 photonic crystal in terms of light modulation is attributed to the light within the red edge region, which can significantly enhance the photocatalytic hydrogen generation efficiency of the photocatalyst.

(1) $$\mathrm{Zn}{\mathrm{In}}_{2}S_{4}+\hbar \nu \to \mathrm{Zn}{\mathrm{In}}_{2}S_{4}\left(e_{c}^{-}+h_{v}^{+}\right)$$

(2) $${3C}_{3}H_{6}O_{3}+{12h}_{v}^{+}\to 3CO_{2}+{12H}^{+}+{3H}_{2}O$$

(3) $${{2H}^{+}+{2e}_{c}^{-}\to H}_{2}$$

**Figure 8:**
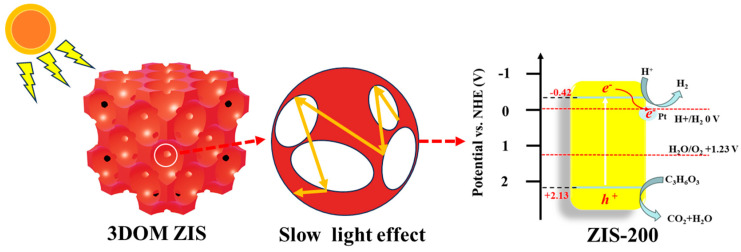
Proposed photolytic mechanism of ZIS–200.

In order to further reveal the mechanism of photocatalysis enhancement of the inverse opal structure for ZIS materials, as shown in Figure 8, in the presence of visible light, the unique structure of ZIS–200 facilitates light propagation within the material and between the material and the air, extending the path of light propagation and enhancing the interaction with light materials. Additionally, the slow light effect of the photonic crystals in ZIS–200 enhances the response of photocatalytic materials to sunlight, thereby significantly improving the efficiency of hydrogen production. Additionally, when the incident photon energy is equal to or greater than the ZnIn_2_S_4_ energy bandgap (E_g_ = 2.55eV) (*hν* ≥ E_g_) and strikes the photocatalyst surface, the valence band electrons (e^−^) move along the band after absorbing the energy of the incident photon, leaving holes (h^+^). Therefore, it produces an electron–hole pair. The reaction between the hole (h^+^) and the lactic acid (C_3_H_6_O_3_) produces carbon dioxide, water and hydrogen ions, as shown in the following Equations (1)–(3). The last two hydrogen ions undergo a half–reaction of hydrogen production under the induction of electrons. In short, 3DOM ZIS can effectively realize charge separation by using its structural characteristics and slow light effect, lengthen the light propagation path and improve the efficiency of the interface charge diffusion process, thus improving the photocatalytic performance. Finally, even though the destruction of the inverse opal structure in the ZIS results in a high specific surface area, enhancing the carrier separation efficiency without a significant impact on the band position of the ZIS, it diminishes the light utilization. Consequently, IO–ZIS demonstrates superior photocatalytic performance compared to B–ZIS, as the inverse opal structure does not provide an advantage in reducing the activation energy of the ZIS, making it essential for efficient light utilization.

## 4. Conclusions

In summary, IO–ZIS photocatalysts were fabricated using the colloidal crystal template method followed by etching to remove the template. Compared to other samples, ZIS–200 shows better photocatalytic performance, in which the hydrogen production rate reaches 14.32 μmol∙m^−2^ h^−1^ with a larger photocurrent intensity and appropriate photonic bandgap, which is 4.4 times that of ZIS–120, 3.4 times that of ZIS–160, 4.8 times that of ZIS–220 and 5 times that of B–ZIS after the specific surface area normalization. The increased photocatalytic activity originated from the slow light effect, which could effectively suppress the recombination of photogenerated charge carriers and greatly improve the light utilization efficiency. According to an analysis of the hydrogen production mechanism, the red edge of the phonic stopband is coupled with the intrinsic absorption edge of the ZIS, which is designed to maximize the slow photon effect, and ultimately improves the absorption efficiency of light. This study offers comprehensive insights into the fabrication of efficient visible light–utilization photocatalysts utilizing photonic crystal materials. This will promote the development of 3DOM structured photocatalytic hydrogen production and provide a new design strategy for the construction of new photocatalysis systems.

## Figures and Tables

**Table 1:** The BET of B–ZIS, ZIS–200, ZIS–160, ZIS–120 and ZIS–220.

Photocatalysts	$\mathrm{BET}\;(m^{2}\cdot g^{-1})$	$\mathrm{Total}\;\mathrm{Pore}\;\mathrm{Volume}\;({\mathrm{cm}}^{3}\cdot g^{-1})$	Average Pore Diameter (nm)
B–ZIS	105.207	0.1698	6.45435
ZIS–200	36.586	0.1561	17.0672
ZIS–160	46.982	0.2196	18.6953
ZIS–120	51.786	0.2359	18.2223
ZIS–220	57.562	0.2223	15.4504

## Data Availability

The research data are available upon demand.

## Supplementary Materials

The following supporting information can be downloaded at: https://www.mdpi.com/article/10.3390/nano14100843/s1. Table S1: Comparison of the photocatalytic hydrogen production rates reported in the literature with those of the prepared IO–ZIS; Figure S1: EDS image of ZIS–200; Figure S2: Element mappings of B–ZIS; EDS image; Figure S3: Hydrogen evolution and hydrogen evolution rate of B–ZIS under different bandpass wavebands. References [42,43,44,45,46,47,48,49,50,51] are cited in the Supplementary Materials.
