# Supplementary material for: Construction of Inverse–Opal ZnIn2S4 with Well–Defined 3D Porous Structure for Enhancing Photocatalytic H2 Production
Source: Nanomaterials (Basel). 2024 May 11;14(10):843. doi: 10.3390/nano14100843 (PMC11123994; doi:10.3390/nano14100843)
Supplement: Supplementary file 1 [file nanomaterials-14-00843-s001.zip › nanomaterials-2950939-supplementary.pdf]

nanomaterials

MDPI

Supplementary Materials

Construction of Inverse–Opal $ZnIn_2S_4$ with Well–Defined 3D Porous Structure for Enhancing Photocatalytic H_2 Production

Yiyi Xie [1,2], Zhaohui Wu [2], Sifan Qi [2], Jiajun Luo [2], Shuang Pi [2], Huanghua Xu [2], Shumin Zhang [2], Difa Xu [2], Shiying Zhang [2,*] and Xianfeng Yang [1,*]

[1] College of Materials Science and Engineering, Changsha University of Science & Technology, Changsha 410114, China
[2] Hunan Key Laboratory of Applied Environmental Photocatalysis, Changsha University, Changsha 410005, China; zsmstudy@foxmail.com (S.Z.)
* Correspondence: cdzhangshiying@ccsu.edu.cn (S.Z.); yangxfcsust@csust.edu.cn (X.Y.)

Table S1. Comparison of the photocatalytic hydrogen production rates reported in the literature with those of the prepared IO-ZIS.

Photocatalyst	Light source	Sacrificial agent	Photocatalyst dosage	Performance (μmol h^{-1} g^{-1})	Ref
the dual-defect Ag-$ZnIn_2S_4$	300W Xe lamp, (λ > 420 nm)	Na_2SO_3 and Na_2S	12 mg	56.6	[1]
Au-PbS-CdS	300W Xe lamp, (λ > 420 nm)	Na_2SO_3 and Na_2S	10 mg	513.63	[2]
$ZnIn_2S_4$@ZnS	300W (λ≥420nm)	Na_2S/ Na_2SO_3	100 mg	2873	[3]
ZIF-derived ZnS/$ZnIn_2S_4$	300 W Xe lamp (1.5G)	TEOA	50 mg	453.4	[4]
TiO_2-$ZnIn_2S_4$	300 W Xe lamp	TEOA	20 mg	214.9	[5]
Time-Dependent synthesis of ZnS	Mercury lamp (λ=254nm)	MetOH	100 mg	276	[6]
CdS@$W_{18}O_{49}$	300W Xe lamp, (λ > 420 nm)	-	100 mg	11732	[7]
Pt/BP/$ZnIn_2S_4$	300W Xe lamp, (λ > 420 nm)	Na_2S/ Na_2SO_3	20 mg	1278	[8]
MoS_2/$ZnIn_2S_4$	300W Xe lamp, (λ > 420 nm)	TEOA	20 mg	221.71 μmol h^{-1}	[9]
CdS@Ni_2P	300W Xe lamp, (λ > 420 nm)	Na_2S/ Na_2SO_3	50 mg	287	[10]
Inverse opal $ZnIn_2S_4$	300W Xe lamp, (λ > 400 nm)	$C_3H_6O_3$	10 mg	14.32 μmol h^{-1}	This work

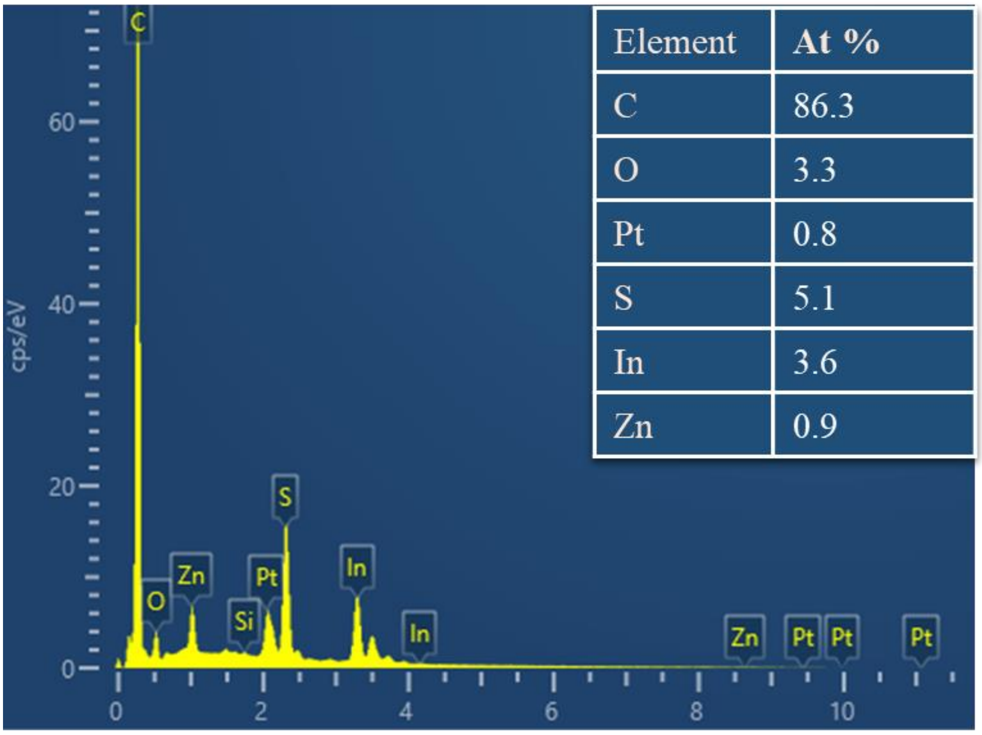

Figure S1. EDS image of ZIS-200.

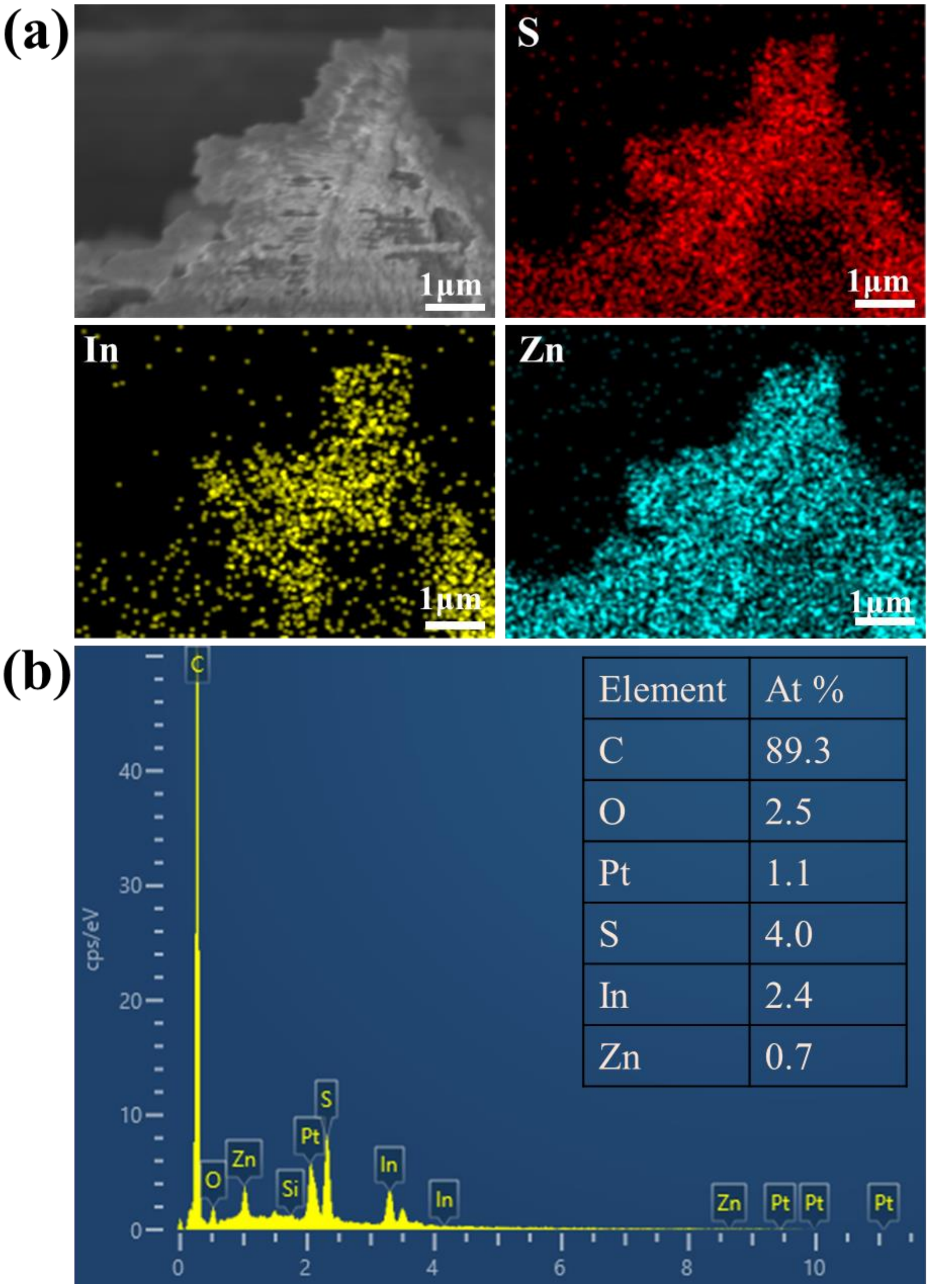

Figure S2. element mappings of B-ZIS (**a**); EDS image (**b**).

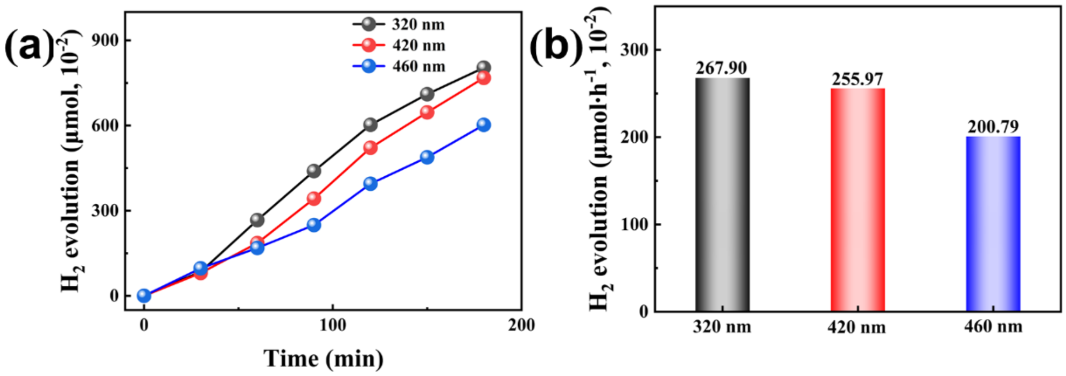

Figure S3. Hydrogen evolution (**a**) and hydrogen evolution rate (**b**) of B-ZIS under different band-pass wavebands.

References

1. Pan, R.; Hu, M.; Liu, J.; Li, D.; Wan, X.; Wang, H.; Li, Y.; Zhang, X.; Wang, X.; Jiang, J.; et al. Two−Dimensional All−in−One Sulfide Monolayers Driving Photocatalytic Overall Water Splitting. *Nano Lett.* **2021**, *21*, 6228–6236. https://doi.org/10.1021/acs.nanolett.1c02008.
2. Chen, W.−B.; Hu, L.−Y.; Meng, F.; Tang, L.; Liang, S.; Li, J.−B. Dual−plasmon−induced photocatalytic performance enhancement in Au−PbS−CdS nanodumbbells with double Au caps on the ends. *Opt. Mater.* **2021**, *117*, 111210. https://doi.org/10.1016/j.optmat.2021.111210.
3. Yuan, X.; Li, P.; Wang, S.; Liu, P.; Zhao, J.; Wang, T.; Chang, K. Facile synthesis of $ZnIn_2S_4$@ZnS composites for efficient photocatalytic hydrogen precipitation. *Catal. Sci. Technol.* **2024**, *14*, 630–637. https://doi.org/10.1039/d3cy01475d.
4. Song, H.; Wang, N.; Meng, H.; Han, Y.; Wu, J.; Xu, J.; Xu, Y.; Zhang, X.; Sun, T. A facile synthesis of a ZIF−derived $ZnS/ZnIn_2S_4$ heterojunction and enhanced photocatalytic hydrogen evolution. *Dalton Trans.* **2020**, *49*, 10816–10823. https://doi.org/10.1039/d0dt02141e.
5. Zuo, G.; Wang, Y.; Teo, W.L.; Xian, Q.; Zhao, Y. Direct Z−scheme TiO_2–$ZnIn_2S_4$ nanoflowers for cocatalyst−free photocatalytic water splitting. *Appl. Catal. B Environ.* **2021**, *291*, 120126. https://doi.org/10.1016/j.apcatb.2021.120126.
6. Ramos−Huerta, L.A.; Aguilar−Martínez, O.; Santes, V.; Tzompantzi Morales, F.J.; Santolalla−Vargas, C.E. Time−Dependent synthesis of ZnS and its influence on photocatalytic hydrogen generation. *Chem. Eng. Sci.* **2024**, *294*, 120067. https://doi.org/10.1016/j.ces.2024.120067.
7. Yang, Y.; Qiu, M.; Chen, F.; Qi, Q.; Yan, G.; Liu, L.; Liu, Y. Charge−transfer−mediated photocatalysis of W18O49@CdS nanotubes to boost photocatalytic hydrogen production. *Appl. Surf. Sci.* **2021**, *541*, 148415. https://doi.org/10.1016/j.apsusc.2020.148415.
8. Zhang, Q.; Zhang, J.; Zhang, L.; Cao, M.; Yang, F.; Dai, W.−L. Facile construction of flower−like black phosphorus nanosheet@$ZnIn_2S_4$ composite with highly efficient catalytic performance in hydrogen production. *Appl. Surf. Sci.* **2020**, *504*, 144366. https://doi.org/10.1016/j.apsusc.2019.144366.
9. Fang, H.; Cai, J.; Li, H.; Wang, J.; Li, Y.; Zhou, W.; Mao, K.; Xu, Q. Fabrication of Ultrathin Two−Dimensional/Two−Dimensional $MoS_2/ZnIn_2S_4$ Hybrid Nanosheets for Highly Efficient Visible−Light−Driven Photocatalytic Hydrogen Evolution. *ACS Appl. Energy Mater.* **2022**, *5*, 8232–8240. https://doi.org/10.1021/acsaem.2c00767.
10. Mamiyev, Z.; Balayeva, N.O. Metal Sulfide Photocatalysts for Hydrogen Generation: A Review of Recent Advances. *Catalysts* **2022**, *12*, 1316. https://doi.org/10.3390/catal12111316.

Disclaimer/Publisher's Note: The statements, opinions and data contained in all publications are solely those of the individual author(s) and contributor(s) and not of MDPI and/or the editor(s). MDPI and/or the editor(s) disclaim responsibility for any injury to people or property resulting from any ideas, methods, instructions or products referred to in the content.
